# Forecasting Future Demand of Nursing Staff for the Oldest-Old in China by 2025 Based on Markov Model

**DOI:** 10.34172/ijhpm.2021.63

**Published:** 2021-06-23

**Authors:** Liangwen Zhang, Shuyuan Shen, Yaqian Guo, Ya Fang

**Affiliations:** ^1^State Key Laboratory of Molecular Vaccinology and Molecular Diagnostics, School of Public Health, Xiamen University, Fujian, China.; ^2^School of Economics, Xiamen University, Fujian, China.; ^3^Institute of Medical Information, Chinese Academy of Medical Sciences, Beijing, China.

**Keywords:** The Oldest-Old, Nursing Staff, Demand Forecasting, Markov Model, China

## Abstract

**Background:** An aging population and an increase in the proportion of disabled elderly have brought an unprecedented global challenge, especially in China. Aside lack of professional long-term care facilities, the shortage of human resource for old-age care is also a major threat. Therefore, this study tries to forecast the demand scale of nursing staff for the oldest-old in 2025 in China servicing as a reference for the development plan of human resource for elderly nursing.

**Methods:** Based on CLHLS (Chinese Longitudinal Healthy Longevity Survey) 2011 and 2014, Logit model was used to construct the transition probability matrix of the elderly’s health status (health/mild/moderate/severe disability and death). By using the data of the elderly population aged 65 or over in the 2010 national population census, we projected the number of Chinese oldest-old population in different health status by 2025 through Markov model and projected the scale of the demand of nursing staff combined with the human population ratio method.

**Results:** The forecast shows that the Chinese oldest-old population is about 52.6 million, among which 46.9 million are healthy, 3.7 million are mild, 0.8 million are moderate, and 1.2 million are severely disabled in 2025. Concurrently, the demand scale of nursing staff will be 5.6 million according to the low standard and 11.5 million according to the high standard. Thus, human resource supply of long-term care is worrying.

**Conclusion:** In 2025, the population size of the Chinese oldest-old will be further expanded, and the demand of care will increase accordingly, leading to a vast gap in the nursing staff. Therefore, it is urgent to build a professional nursing staff with excellent comprehensive quality and reasonable quantity, to ensure the sustainable development of China’s elderly care service industry.

## Background

**Key Messages**

**Implications for policy makers**

- Estimating aggregate nursing staff requirements for a country could produce realistic projections to guide investments, recruitment, deployment and retention strategies.
- In 2025, Chinese oldest-old population is about 52.6 million, among which 46.9 million are healthy, 3.7 million are mild, 0.8 million are moderate, and 1.2 million are severely disabled. Policy-makers should create a favorable policy environment for the pension industry.
- Policy-makers in China should give priority to talent introduction of nursing staff in terms of addressing the serious specialists’ shortage.

**Implications for the public**

This paper focuses on projecting the number of the oldest-old population in different health status and the demand of nursing staff for evidence-informed planning in China. On one hand, such evidence would be conducive to assisting the elderly to obtain professional care and reducing care burden of families to improve the quality of life and the happiness index for the elderly. On the other hand, it could be beneficial to optimize the allocation of care resources and improve the quality of nursing homes, and then promote the sustainable development of the China’s elderly care service industry. A talent training system is needed to shape the policy agenda for the professionalism of China’s elderly care service industry.

The aging population has become a serious social problem around the world, especially in China, which is a developing country with the largest population in the world and aging rapidly.^1,2^ China’s aging problem is highlighted in three characteristics, including immense scale, rapid development and the larger oldest-old population.^3^ With the increase of age and degenerative diseases, the body functions of the elderly, especially the oldest-old, such as the functions of various organs and tissues, the ability to move, will decline. Even some older people will lose the ability to live independently. It means that they need to be cared for by their families and society for the rest of their lives. At present, the oldest-old population accounts for about one-tenth of the total elderly population in China, but it is growing at a fast rate of 3.8% per year, higher than other age groups.^4^ It forecasts the oldest-old population aged 80 or above in China will snowball from 2.7 million in 2020 to more than 100 million in 2050.^5^ In the future, the demand for the oldest-old care will increase sharply and China’s old-age security system will face severe challenges.^6^

 In the face of the contradiction between supply and demand in the field of aged care in China, on the one hand, informal family care is under enormous pressure due to the miniaturization of family structure and the high rate of women’s labor participation.^7,8^ On the other hand, formal institutional care also has many problems. For example, the formal institutional care model faces an imbalance between supply and demand.^9^ Likewise, there are still many problems in Chinese nursing homes, such as the lack of nursing professionals,^10^ unreasonable allocation ratio, low degree of specialization, and poor stability of the team.^11,12^ In response to the contradiction between supply and demand, the Chinese government has issued a series of policies in recent years, attaching great importance to building a team of professional nursing assistants with life care and medical care capabilities. Additionally, the Chinese government has continued to improve the long-term care system where ‘home-based care is the foundation, community-based care provides the necessary support and residential care is supplementary.’^13^ Therefore, in the context of the current strategy of combining medical care with nursing care, it is of high policy significance to understand the demand scale of nursing staff for the oldest-old.^14^ By accurately forecasting the demand scale, we can provide a theoretical basis for the scientific construction of professional nursing assistants.

 In China, research on long-term care generally focuses on the demand side, that is, the demand-oriented research, mainly focusing on several provinces, cities, or regions^15^. In the demand forecasting of domestic long-term care, the primary research method is the Markov model.^15^ Generally, the Activity of Daily Living scale and the Instrumental Activity of Daily Living scale are used to classify the health status of the elderly, and the Markov model is used to describe the health status transition change of the elderly and to measure the disability rate of the elderly. Finally, combined with population prediction results, the number of the oldest-old in different years and different disability states is estimated and predicted. Based on the national survey of aged services, the demand for aged care services is estimated and predicted. Zhou used this method to predict China’s elderly caregivers’ demand in 2030, which was about 105 million in the high-level condition, 50 million in the medium level condition, and 35 million in the low-level condition.^16^ Multi-agent model,^17^ random forest,^18^ and other research methods are also used for prediction analysis. The accuracy of these prediction models still needs to be further explored.

 However, research on the prediction of long-term care needs is relatively early in the developed countries. As early as 1986, Bishop found that the cost of long-term care in the United States would increase to 166.2 billion dollars in 2050 by establishing a long-term care prediction model.^19^ In 1998, the London School of Economics and Political Science also predicted that the long-term care needs of the elderly would increase in the future.^20^ Foreign scholars have applied the Markov model to the prediction of aged care demand many times. For example, Pritchard used the tracking data from 1982 to 1984 in the American Long Term Care Survey to calculate the health status transfer matrix of the elderly aged 65 or over by using the Markov model and then predicted the future demand for aged care in the United States.^21^ At present, foreign research on long-term care prediction has become mature. In addition to the Markov model, Wittenberg used the PACSim dynamic microsimulation model to estimate the number of older people with dementia receiving unpaid care or using care services and associated costs in England.^22^ The results showed that the number of older people with dementia would more than double in the next 25 years, and the number receiving unpaid or formal care would be projected to rise by 124%, from 530 000 in 2015 to 1 183 000 in 2040.^22^

 In so doing, this study will improve the research object, research scope, and research method in the future. We sought to address the following questions: (1) How does the health status of the elderly change? (2) What is the scale of the oldest-old in different health status? (3) What is the demand for nursing assistants for the oldest-old? To address the above problems, this study adopted the CLHLS (Chinese Longitudinal Healthy Longevity Survey) data in 2011 and 2014. To begin with, logit model was used to calculate the probability matrix of the health status transition of the elderly from 2008 to 2011. Then the reliability of the Markov model was verified by comparing the survey data of the CLHLS 2014 with the prediction results of the logit model. Next, based on the data of the sixth China population census in 2010, the Markov model was used to predict the population size of the oldest-old aged 80 or above in China in 2025 with different health conditions. To end with, in the light of the allocation ratio of elderly nursing assistants in many provincial and municipal nursing homes, the demand scale of nursing assistants was estimated by the human population ratio method, so as to obtain the forecast result of the scale of the demand for nursing assistants in 2025. Above all, it provides an evidence-based basis for Chinese policy-makers to optimize the allocation of nursing assistants.

## Materials and Methods

###  Data Sources

####  Database

 The data were obtained from the CLHLS in 2008, 2011 and 2014, as well as the data from the sixth national census in 2010. The survey scope of the CLHLS covers 23 provinces, municipalities, and autonomous regions in China. It includes a population-based, nationally representative sample of old Chinese adults and adopts a targeted random-sample design to ensure representativeness, so it has been used to estimate the prevalence of disability among old adults in China, suggesting its good representativeness.^23,24^ We chose respondents aged 65 or above who would be aged 80 or above in 2025 to reflect the oldest-old nursing staff’s demand.

####  The Definition of the Health Status of the Elderly

 The Activity of Daily Living scale adopted in this study refers to the Health and Retirement Study in the United States. Among them, the Basic Activity of Daily Living scale includes six items: bathing, getting dressed, toileting, eating, transferring, and continence. In line with the degree of disability classification standards of the National long-term Care Survey, transferring the elderly health status into healthy (I), mild disability (II), moderate disability (III), severe disability (IV) and death (V). Health status I, II, III, and IV are transition status, and V is the absorption state (death) (Supplementary file 1, Table S1).

###  Model Structure and Assumptions

####  Multinomial Logit Model

 Assuming that the health status of the elderly may be y=1,...*j*, where *j* is a positive integer, there are *j* kinds of mutually exclusive choices. Using the random utility method, the expression of the multinomial logit model is written as follows:


$$P\left(y_{i}=j\left|X_{i}\right.\right)=\exp\left(X_{i}\beta_{i}\right)/\sum_{k=1}^{j}\exp\left(X_{i}\beta_{i}\right)$$

Where, explanatory variables *X*_i_ only change with an individual *i*, not with health status *j*. The coefficients *β*_i_ indicate that the effect on the random effect depends on the health status *j*.

 Since the sum of the probabilities of various health status is 1, the probability that the health status of individual *i* is *j* is:


$$P\left(y_{i}=j\left|X_{i}\right.\right)=\left\{\begin{array}{l} \frac{1}{1+\sum_{k=2}^{j}\exp\left(X_{i}\beta_{i}\right)}\;\left(j=1\right)\\ \frac{\exp\left(X_{i}\beta_{i}\right)}{1+\sum_{k=2}^{j}\exp\left(X_{i}\beta_{i}\right)}\;\left(j=2,...,J\right) \end{array}\right.$$

Where, “*j*=1” is the reference scheme, and this model is called multinomial logit model, which is estimated by maximum likelihood method.

####  Markov Model

 Many statistical models can be used to predict human resources for health, including grey model GM(1,1), autoregressive mean mixed model, regression model, Markov chain process, and so on. Zhou^16^ applied the Markov model to predicting elderly population size and human resources of elderly care and further verified it with the latest survey data of CHARLS (China Health and Retirement Longitudinal Study) in 2015. The results showed that the Markov model was suitable for predicting the size of the elderly population and the human resources of nursing care in China with high accuracy. Markov chain is a set of discrete random variables with Markov properties. Its future state is only related to the present state and has nothing to do with the former state, that is, Markov chain satisfies obliviousness and stationarity. Considering that development process of the elderly’s incapacitated state has the characteristics of time continuity and countability, we used the continuous-time homogeneity Markov process to depict the changes of the elderly’s health status based on the data modeling of 2008 and 2011 in CLHLS. Then, multinomial logit model was used to calculate the transfer probability, and the transfer probability matrix between different health conditions was established by age group, to track the health evolution trajectory in the life cycle of the elderly, and then to calculate the number of the elderly with different health conditions in different age groups (Figure). Meanwhile, the hypothesis of this study is as follows: age-specific morbidity and disease structure of the elderly should remain the same, medical technology and preventive care standards should remain the same, and patient examination behavior should remain the same.

**Figure:**
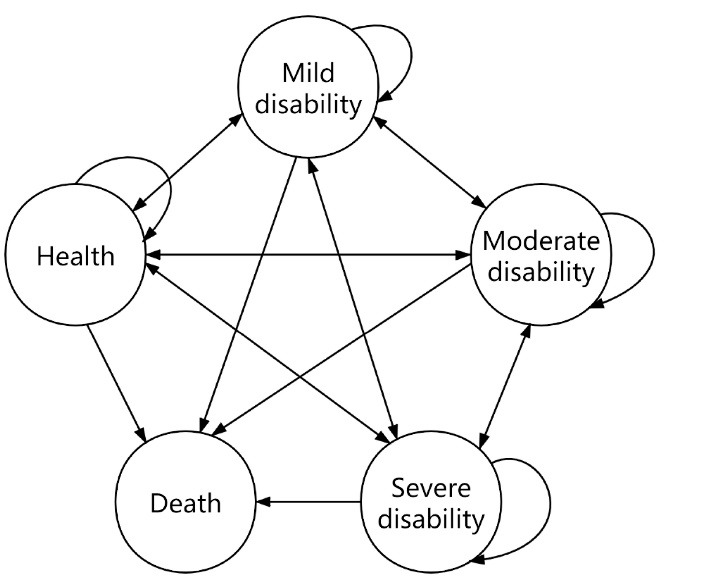
The Markov Transition Model Among the Five Health Status of the Elderly.

 Considering the random and mutually exclusive nature of the health status transfer, it is necessary to use probability to describe the possibility of the health status transfer. The probability of the transfer from health status *i* to health status *j* is denoted as:


$$P_{ij}=P\left(E_{i}\left|E_{j}\right.\right)=P\left(E_{i}\to E_{j}\right)$$

Among them, the health status transition probability has the following characteristics:


$$0\leq P_{ij}\leq 1\;\sum_{j=1}^{k}P_{ij}=1,\;i,j=1,2,...,k$$

Under hypothesis certain condition, the system can only transfer to each other in a possible state of health, such as *P*_i1_, *P*_i2_,… *P*_ik_ (*i*=1,2,…,*k*), and its health status transition probability matrix is as follows:


$$\left\{\begin{array}{l} P_{11}\;P_{12}\;...\;P_{1k}\\ P_{21}\;P_{22}\;...\;P_{2k}\\ P_{31}\;P_{32}\;...\;P_{3k}\\ \;...\;...\;...\;...\\ P_{k1}\;P_{k2}\;...\;P_{kk} \end{array}\right\}$$

The WinBUGS software 1.4 is used to perform Markov simulation, where the refresh and updates of the model are set to 100 and 2000 respectively.

####  Human Population Ratio Method

 The human population ratio method is one of the four classical health workforce measurement methods recommended by The World Health Organization (WHO).^25^ It can be used to predict both the demand and supply of the health workforce. The calculation method is as follows: future health workforce demand = ratio of the workforce population in the target year × the number of target year population. We applied it to calculate the demand for the nursing assistants for the oldest-old.

####  Sensitivity Analysis

 Uncertainty around the point estimates of nursing staff requirements arising from parameter uncertainty, particularly the transition probabilities are explored by simultaneously varying all the transition probabilities in the predictive model using both their lower and upper 95% confidence limits. In addition, previous studiespointed out that uncertainty could be expressed as a predictive interval, providing the upper and lower boundaries of a range within which the observed value was expected.^26^

###  Data Analysis and Processing

 We used Stata 15.0 for data cleaning and statistical description, described transfer probability between different health status of the elderly by multinomial logit models, and evaluated the predictive efficacy of the Markov model by chi-square test. Afterward, Matlab 2017 software was used to calculate the transfer probability between health conditions based on the Markov model. Ultimately, the size of the oldest-old population with different health conditions in China in 2025 was predicted.

## Results

###  Basic Information

####  Basic Information of the Research Object

 In this study, a total of 13 269 respondents were finally included who participated in two follow-ups CLHLS surveys in 2008 and 2011 and were aged 65 or above in 2008. Of these subjects, 7614 women accounted for 57.38%, and 61.98% of the elderly aged 85 or above were included (Table 1).

**Table 1:** Basic Information of the Research Object

	**Number**	**Percent**
Gender		
Male	5655	42.62
Female	7614	57.38
Age (y)		
65-69	1111	8.37
70-74	1223	9.22
75-79	1118	8.43
80-84	1593	12.01
≥85	8224	61.98

####  Distribution of Study Subjects’ Health Status in 2008 and 2011

 In 2008, there were 10 406 healthy older people, accounting for 78.42% of the total population. There were 1461, 683 and 719 older people with mild, moderate, and severe disabilities, accounting for 11.01%, 5.15% and 5.42%. In 2011, there were 5767 healthy older people (73.48%); there were 1086, 383 and 612 older people with mild, moderate and severe disabilities, accounting for 13.84%, 4.88% and 7.80% respectively (Table S2).

###  Construct the Health Status Transition Probability Matrix

####  Changes in the Subjects’ Health Status From 2008 to 2011

 In 2011, 5851 older people maintained the same health status, and 933 changed from healthy to mild disabled people, and 269 turned from healthy to moderate disabled people, and 382 turned from healthy to severely disabled people, and 3517 turned from healthy to dead people (Table S3).

####  Distribution of Health Status Transfer Probability Between 2008 and 2011

Table 2 shows the health transfer probability matrix of the research subjects from 2008 to 2011. Under the same health condition, the younger the age is, the higher the probability of health transfer to health is. Conversely, the older the person is, the more likely he or she is to transfer health status to death. The healthier the subjects were in the same age group, the less likely they were to move to the end. On the contrary, the worse the health status is, the higher the probability of death transfer is.

**Table 2:** Distribution of the Health Status of Study Subjects Transfer Probability From 2008 to 2011

**Health Status in 2008**	**Health Status in 2011**					
	**Age (y)**	**Health**	**Mild Disability**	**Moderate Disability**	**Severe Disability**	**Death**
Health	65-69	0.8982	0.0444	0.0061	0.0052	0.0461
	70-74	0.8283	0.0505	0.0140	0.0117	0.0956
	75-79	0.7425	0.0675	0.0124	0.0195	0.1581
	80-84	0.6395	0.0866	0.0199	0.0301	0.2239
	≥85	0.3293	0.1039	0.0341	0.0501	0.4826
Mild disability	65-69	0.4000	0.5000	0.0000	0.0000	0.1000
	70-74	0.5652	0.1739	0.0435	0.0000	0.2174
	75-79	0.3696	0.1739	0.0652	0.0652	0.3261
	80-84	0.3295	0.1591	0.0227	0.1364	0.3523
	≥85	0.0909	0.1114	0.0499	0.0755	0.6723
Moderate disability	65-69	0.6000	0.0000	0.0000	0.4000	0.0000
	70-74	0.2500	0.1250	0.0000	0.0000	0.6250
	75-79	0.0833	0.0833	0.1667	0.2500	0.4167
	80-84	0.1111	0.0741	0.0741	0.2963	0.4444
	≥85	0.0405	0.0358	0.0576	0.1028	0.7632
Severe disability	65-69	0.0000	0.0000	0.0000	0.2500	0.7500
	70-74	0.1875	0.0625	0.0625	0.1250	0.5625
	75-79	0.0714	0.0000	0.0000	0.2857	0.6429
	80-84	0.0313	0.0313	0.0313	0.1875	0.7188
	≥85	0.0296	0.0178	0.0178	0.0607	0.8743

####  Logit Model Calculated the Probability Distribution of Health Status Transfer From 2008 to 2011

 Using the logit model, the health status in 2011 was taken as the dependent variable and the health status in 2008 as the independent variable. After the age factor was included, we obtained the health transfer probability matrix of the research objects from 2008 to 2011. Based on the same health status, the younger the age is, the higher the probability of health transfer to health is. Conversely, the older the person is, the more likely he or she is to transfer health status to death. Otherwise, it was by removing some extreme values that the model was adjusted to make the health status transition probability more realistic and reliable (Table S4, Figures S1-S4).

###  Verification of the Reliability of the Health Status Transfer Probability Matrix

####  Distribution of CLHLS 2014 Subjects With Different Health Conditions in Different Age Groups

 The screening criteria of research objects in the CLHLS 2014 are as follows: (1) Basic Activity of Daily Living questions have valid answers and no missing values; (2) Aged 65 or above. The sample size that was finally included in this study was 7107. Of the research objects, females were 3856 (54.26%), the elderly aged 85 or above were 3662, and the healthy elderly accounted for 76.7% (Table S5).

####  Health Status Distribution of the Elderly Population Aged 65 or Above in China in 2010

 According to the 2010 population census data and the health status transfer probability measured by CLHLS from 2008 to 2011, we estimated the health status distribution of the elderly population aged 65 or above in China in 2010. What’s more, it was estimated that the healthy elderly population was about 107.3 million, and the moderate, moderate, and severe elderly population were 6.4, 2.2, and 2.9 million, respectively (Table S6).

####  Comparison of Health Transfer Probability Prediction Results and CLHLS 2014 Survey Results

 In the light of the health status transfer probability matrix calculated by logit model and Markov model, combined with the health status distribution data of the elderly population in China in 2010 in Table S7, the health status distribution data of the older people aged 65 or above in China by 2014 was estimated. Chi-square test was used to verify whether the predicted results were different from actual CLHLS 2014 results. It was shown that χ^2^=7.487, *P*> .05, no statistical difference between them, namely healthy transition probability matrix obtained through the logit model was reasonable to predict the elderly health status (Table S7).

###  Prediction Results of the Size of the Oldest-Old Population With Different Health Conditions in China in 2025

 According to the health transition probability matrix in 2008-2011, and the health status of China’s older people aged 65 or above in 2010, it was predicted that the population of the oldest-old aged 80 or above would be 52.6 million in China by 2025, including about 46.9 million healthy people, 3.7 million mild disabled people, 0.8 million moderate disabled people, and 1.2 million severe disabled people (Table 3).

**Table 3:** Prediction Results of the Scale of the Oldest-Old Population With Different Health Conditions in China in 2025

**2010 Census Data**	**Health Status in 2025**					
**Health Status**	**Age (y)**	**Health**	**Mild Disability**	**Moderate Disability**	**Severe Disability**	**Death**
Health	65-69	7 692 304	460 913	63 982	82 667	2 702 410
	70-74	6 516 655	390 727	54 276	70 189	3 982 432
	75-79	4 969 719	298 045	41 414	53 579	5 639 833
	80-84	3 764 901	225 744	31 361	40 561	6 939 502
	≥85	5 332 706	384 655	118 392	103 581	5 062 084
Mild disability	65-69	3 863 268	278 958	86 007	75 366	6 698 205
	70-74	2 518 096	181 946	56 166	49 274	8 207 706
	75-79	1 696 838	122 549	37 800	33 138	9 111 397
	80-84	3 172 742	350 224	82 962	152 855	7 244 418
	≥85	1 975 507	218 459	51 892	95 866	8 672 995
Moderate disability	65-69	1 137 944	125 968	29 978	55 491	9 641 891
	70-74	726 502	80 378	19 112	35 348	10 141 280
	75-79	1 660 797	278 237	72 782	165 155	8 796 686
	80-84	912 627	153 428	40 338	92 009	9 795 322
	≥85	493 659	83 166	21 946	50 260	10 349 016
Severe disability	65-69	302 648	50 940	13 424	30 701	10 602 185
	70-74	74 132	31 982	13 883	23 769	10 858 247
	75-79	30 717	13 380	5881	10 174	10 931 369
	80-84	14 864	6505	2881	5019	10 974 736
	≥85	8075	3525	1556	2703	10 998 968
Total		46 864 699	3 739 730	846 033	1 227 704	167 350 681

###  Allocation Ratio of Nursing Assistants for the Oldest-Old

 We selected the allocation ratio requirements of nursing assistants and older people in nursing homes from some representative provinces and cities in China, summarized as follows (Table S8).

 As nursing assistants’ allocation ratio for the oldest-old had not been stipulated in various places, we mainly referred to the allocation standard for all older people in the policy document. The expert consultation method was used to discuss and integrate these allocation ratios. Eventually, the allocation ratio of nursing assistants and older people was finally set to low and high levels (Table S9).

###  The Demand Scale of Nursing Assistants for the Oldest-Old in 2025

 Combined with Table S8 and Table S9, we could forecast the demand for nursing assistants for the oldest-old in China in 2025 through the human population ratio method. For nursing assistants of the oldest-old in China by 2025, according to the low level, the need is about 5.6 million; while according to the high level, the need is about 11.5 million (Table 4).

**Table 4:** Forecast Results of the Demand Scale for Nursing Assistants for the Oldest-Old in China in 2025 (Million)

	**Health**	**Mild Disability**	**Moderate Disability**	**Severe Disability**	**Total (95% CI)**
The oldest-old population	46.86	3.74	0.85	1.23	52.68 (49.35,58.24)
Low level	4.69	0.47	0.14	0.31	5.61 (5.13,6.45)
High level	9.38	0.93	0.34	0.82	11.47 (9.76,14.62)

## Discussion

 With the increase of life expectancy and the improvement of medical conditions, the aging population has become a significant challenge facing China and even the whole world. Studies have shown that the oldest-old, especially those who are disabled, empty nests or living alone, are more willing to receive professional care services in long-term care institutions, so it is particularly important to predict the demand of nursing assistants for the oldest-old.^27,28^ In this study, the national representative longitudinal survey data and the Markov model are used to reflect the sample information from the individual level fully, and calculate the number of nursing assistants needed by the oldest-old in China, in order to provide baseline data and scientific basis for the government to formulate a reasonable aging strategy.

 In this study, it is found that among study subjects in the same health status, the ones aged younger are more likely to maintain their current situation or recover to better health status as well as a lower mortality rate than those aged older. The transition probability of health status for older people at different age groups is different, but the changing trend is same, that is, the worse the health status is, the more difficult it is to recover back to self-care state or the better health status. As health deteriorates, so does death rate. The possible reason is that with the increase of age, physiological function decreases, health problems increase, health status deteriorates, recovery ability weakens, the probability of different levels of disability increases, and the risk of death increases. This changing trend is also consistent with the research results obtained by other scholars.^11,16,29-32^ The above results suggest that the probability of maintaining health and the likelihood of improving health are relatively high for the mildly disabled older people. Therefore, health management, prevention, and intervention should be focused on prolonging the healthy life of them. For the elderly with moderate and severe disabilities, the focus should be on medical and health services and the construction of professional and standardized service teams. Meanwhile, these results are also conducive for the government and relevant departments to formulate policies dealing with the disability of the elderly, aiming to delay the deterioration and pay more attention to the most vulnerable groups with severe disability.

 The prediction results show that in 2025, there will be 52.6 million older people aged 80 or above in China, among which there will be 46.9 million healthy older people and 3.7, 0.8, and 1.2 million older people with mild, moderate and severe disabilities, respectively. At the same time, in 2025, the demand scale of nursing assistants for the oldest-old will reach 0.56 million according to the low standard, while 1.15 million according to the high standard. It reflects that China’s elderly population base is vast, and the aging process is accelerating. The oldest-old are in poorer health, with a higher disability rate and greater demand for nursing assistants. The shortage of nursing assistants will become more severe in the future. Also, according to the 2017 China Civil Affairs Statistics Yearbook, by the end of 2016, there was less than 20 thousand certified nursing staff in China, which indicated that there would be a massive gap in China’s nursing staff in the future, and the supply of human resources for elderly care would be worrying.

 The research of foreign scholarsalso reflects the increasing demand of the elderly for professional nursing care.^33-35^ Kingston’s study showed that with more people aged 85 or above in the United Kingdom in the next 20 years, its dependencies, dementia, and complications would reach a higher level. So the social care service must adapt to the growing aging population with complex care needs, especially the demand of the oldest-old. Freedman analyzed the 2011 National Health and Aging Trends Study and examined activity constraints and assistance, care resources, and unmet needs of a national sample of the elderly.^36^ They also found that the elderly population, especially those with few financial resources, has significant post-care needs. Jagger et al predicted the nursing needs of the elderly aged 85 or over in Newcastle in the future, finding that the number of people needing 24-hour care would increase substantially, and the demand for nursing homes would increase correspondingly.^37^

 Therefore, training nursing personnel for the aged is of vital importance.^38^ It should multi-directionally develop training channels to stimulate the growth of the elderly nursing personnel from the government, the market, the society and other different training modes, according to the different subjects of responsibility, operation mechanism and operation conditions. Concurrently, the nursing staff should be managed at a multi-level.^39^ Through subsidies, the provision of the minimum salary and other forms to improve the salary level of front-line workers, more young people will be attracted to engage in the elderly care industry.^40^ Then a solid foundation will be laid for future diversified multi-level elderly care services.^41^ While expanding the number of nursing assistants, it should also pay attention to the quality and scientific development of nursing assistants. The Ministry of Education should set up majors related to “elderly business management,” to encourage colleges and universities to set up related majors in undergraduate courses. Formulating strict requirements and norms for personnel training helps to form a systematic professional personnel training system for elderly service and improve the overall quality of nursing assistants.^42^ With the diversification of the pension service demand and the surge in demand,^43-45^ the Chinese government is difficult to meet the various demand of the elderly. It needs to lead the capital into the pension industry science and allocate resources reasonably so that the development of home endowment, community endowment, and institution endowment can be developed healthily. Finally, a scientific and diversified service system for the aged will be established.

###  Strengths and Limitations

 At the current stage, the aging process is prominent, and the disability rate of the oldest-old is higher than that of other age groups. Still, there is a lack of scale prediction of nursing staff for this group. It is obvious that this study has both content and perspective innovation. Furthermore, traditional statistical methods, such as Logistic regression and Cox regression, can only study the changes between two health conditions. Accordingly, based on the National Longitudinal Survey, we not only use multi-state Markov model to conduct continuous dynamic research on stochastic process but also consider other factors such as health status, outcomes and transfer time between health status, as well as the complex situation of various health status transferring and continuously developing, to make the forecast results more scientific and accurate.

 However, there are still shortcomings. Firstly, the academic community has not unified the definition of the elderly’s health status, especially the description of the care standards for the oldest-old. This study refers to Tao’s study^46,47^ and the Katz index^47^ classification method to classify the health status of the elderly. If different ways are used to classify health conditions, the results may be skewed. Secondly, there is still a lack of standardized allocation ratio of nursing assistants for the oldest-old, and the final results may be biased to some extent. Thirdly, this study did not consider the weight of the database, which may lead to the deviation of the predicted results. Therefore, we will further explore the proportion of the required configuration under the community, through the Delphi method, to enhance its authority in future studies. On the other hand, we will combine with other databases to make predictions and the weight of the database is considered. For instance, we will use the data of middle-aged (≥45 years) to calculate the demand scale of nursing assistants for older people aged 65 or above with different health conditions.

## Conclusion

 In summary, the population size of the oldest-old in China will be further expanded in 2025, and the demand for care will increase accordingly, leading to a vast gap in nursing assistants for the elderly. The increase in the oldest-old population directly leads to an increase in the demand for nursing assistants, which is 5.6 million at the low standard and 11.5 million at the high standard. Considering the shortage of nursing staff and the low overall professional level in China, the contradiction between supply and demand will be further prominent in 2025. Therefore, policy-makers currently need to formulate a comprehensive plan for the training of elderly nursing staff in terms of education, training, salary and promotion as soon as possible.

 Firstly, it is necessary to establish a talent training system for elderly care service, which takes vocational education as the main body and pays equal attention to academic education and vocational training. Secondly, it is urgent to improve the entrance examination and evaluation standard system. It should strictly control the professional level of nursing staff and supervise the management standard of long-term care institutions. Thirdly, it should clarify the service items and standardize the number of nursing staff, which are needed by the elderly in different disabled states, so as to realize the rational utilization and optimization of human resources. Last but not least, the government should also do a good job in education, publicity, and promotion of the senior service industry, and change the stereotype of the society for the senior service industry, so as to create a professional and reasonable number of nursing staff for the country, and ensure the healthy and sustainable development of the pension service system.

## Acknowledgements

 The authors would like to thank the Healthy Ageing and Development Research Center at Peking University for its support with the data. We are also grateful to the students in the School of Public Health, Xiamen University who participated in the data collation and analysis.

## Ethical issues

 The study did not involve human subjects and care was taken to ensure anonymity such that no identifiable information of individual nursing home, patient or staff is reported.

## Competing interests

 Authors declare that they have no competing interests.

## Authors’ contributions

 LZ, SS, YG, and YF worked together. LZ taked the study design, analyzed and interpreted the data, and drafted the manuscript. SS and YG participated in drafted the manuscript. YF supervised and critically revised the manuscript. All authors have read and agreed to the published version of the manuscript.

## Funding

 This study was supported by the China Postdoctoral Science Foundation (grant number 2020M671949), and the National Natural Science Foundation of China (grant number 81973144). The funders who supported this study had no role in study design, data collection and analysis, decision to publish, or preparation of the manuscript.

## Supplementary files

Supplementary file 1 contains Tables S1-S9 and Figures S1-S4.
