# Supplementary material for: Forecasting Future Demand of Nursing Staff for the Oldest-Old in China by 2025 Based on Markov Model
Source: Int J Health Policy Manag. 2021 Jun 23;11(8):1533–41. doi: 10.34172/ijhpm.2021.63 (PMC9808353; doi:10.34172/ijhpm.2021.63)
Supplement: Supplementary file 1 — contains Tables S1-S9 and Figures S1-S4. [file ijhpm-11-1533-s001.pdf]

Article title: Forecasting Future Demand of Nursing Staff for the Oldest-Old in China by 2025 Based on Markov Model

Journal name: International Journal of Health Policy and Management (IJHPM)

Authors' information: Liangwen Zhang[1,2], Shuyuan Shen[1], Yaqian Guo[3], Ya Fang[1]*

[1]State Key Laboratory of Molecular Vaccinology and Molecular Diagnostics, School of Public Health, Xiamen University, Fujian, China.

[2]School of Economics, Xiamen University, Fujian, China.

[3]Institute of Medical Information, Chinese Academy of Medical Sciences, Beijing, China.

(*Corresponding author: fangya@xmu.edu.cn)

Supplementary file 1

Table S1. Criteria for the classification of health status of the elderly

Health status	Classification standard
Health（I）	Non-disabled
Mild disability（II）	1~2 BADL Dependencies
Moderate disability（III）	3~4 BADL Dependencies
Severe disability（IV）	5~6 BADL Dependencies
Death（V）	Death

Table S2. Distribution of subjects' health status from CLHLS 2008 and 2011 in 2008

Health status	2008		2011	
	Number(N)	Percentage(%)	Number(N)	Percentage(%)
Health	10406	78.42	5767	73.48
Mild disability	1461	11.01	1086	13.84
Moderate disability	683	5.15	383	4.88
Severe disability	719	5.42	612	7.80

Table S3. Changes in the study subjects' health status from 2008 to 2011

Health status in 2008	Health status from 2008 to 2011					
	Health	Mild disability	Moderate disability	Severe disability	Death	Total
Health	5851	933	269	382	3517	10952
Mild disability	187	183	74	118	969	1531
Moderate disability	35	27	41	79	512	694
Severe disability	25	14	14	54	635	742
Total	6098	1157	398	633	5633	13919

Table S4. Logit model calculated the health status transition probability distribution of study subjects from 2008 to 2011

Health status in 2008	Health status in 2011					
	Age（year）	Health	Mild disability	Moderate disability	Severe disability	Death
Health	65~69	0.895	0.047	0.006	0.007	0.045
	70~74	0.829	0.052	0.014	0.011	0.094
	75~79	0.740	0.069	0.014	0.023	0.154
	80~84	0.641	0.087	0.019	0.037	0.215
	≥85	0.330	0.103	0.034	0.047	0.486
Mild disability	65~69	0.653	0.138	0.021	0.030	0.159
	70~74	0.509	0.128	0.044	0.04	0.279
	75~79	0.377	0.141	0.038	0.068	0.377
	80~84	0.274	0.15	0.042	0.091	0.443
	≥85	0.093	0.117	0.049	0.077	0.663
Moderate disability	65~69	0.475	0.079	0.045	0.077	0.324
	70~74	0.306	0.061	0.078	0.085	0.471

	75~79	0.198	0.058	0.059	0.128	0.556
	80~84	0.131	0.057	0.06	0.156	0.596
	≥85	0.038	0.037	0.059	0.112	0.754
Severe disability	65~69	0.376	0.049	0.019	0.064	0.492
	70~74	0.220	0.034	0.029	0.065	0.652
	75~79	0.134	0.031	0.021	0.091	0.723
	80~84	0.086	0.029	0.021	0.108	0.756
	≥85	0.023	0.018	0.019	0.071	0.870

Table S5. Distribution of subjects with different health conditions in different age groups from CLHLS 2014

	Number (N)	Percentage (%)
Gender		
Male	3251	45.74
Female	3856	54.26
Age (year)		
65~69	226	3.18
70~74	950	13.37
75~79	1193	16.79
80~84	1076	15.14
≥85	3662	51.53
Health status		
Health	5451	76.70
Mild disability	838	11.79
Moderate disability	331	4.66
Severe disability	487	6.85

Table S6. Health status distribution of the elderly population aged 65 or above in China in 2010

Health status	Age group（year）	Number
Health	65~69	38,748,359
	70~74	31,132,884
	75~79	21,173,017
	80~84	11,384,618
	≥85	4,897,539
Mild disability	65~69	1,091,503
	70~74	1,110,649
	75~79	1,739,426
	80~84	1,155,862
	≥85	1,283,379
Moderate disability	65~69	545,752
	70~74	347,078
	75~79	439,855
	80~84	310,716
	≥85	560,961
Severe disability	65~69	727,669
	70~74	381,786
	75~79	499,835
	80~84	522,002
	≥85	838,336

Table S7. Comparison of health transfer probability prediction results and CLHLS 2014 survey results

	Health	Mild disability	Moderate disability	Severe disability	χ^2	df	P
Survey results	76.97%	11.55%	4.60%	6.77%	7.487	3	0.06
Prediction results	75.54%	13.08%	4.43%	6.94%			

Table S8. Requirements on the ratio of nursing assistants to the elderly in nursing homes in more than ten provinces and cities

	Shanghai	Guangdong	Henan	Hangzhou	Wuxi	Suzhou	Beijing	Jinan	Hubei	Ningxia	Shanxi
Health	1:5-1:10	1:10	1:10	1:8-1:14	1:8-1:10	1:10	1:7	1:7	1:6	1:10	1:6
Half disability	1:2.5-1:3.5	1:6	1:5	1:4-1:8	1:5-1:8	1:5-1:6	1:5	1:5	1:4	1:5-1:8	1:4
Severe disability	1:1.5-1:3.5	1:3	1:3	1:1-1:5	1:3-1:5	1:3	1:3	1:3	1:3	1:3	1:3

Table S9. Allocation ratio of nursing assistants and old people

Type	Low level	High level
Nursing assistants: healthy old people	1:10	1:5
Nursing assistants: mild disabled old people	1:8	1:4
Nursing assistants: moderate disabled old people	1:6	1:2.5
Nursing assistants: severe disabled old people	1:4	1:1.5

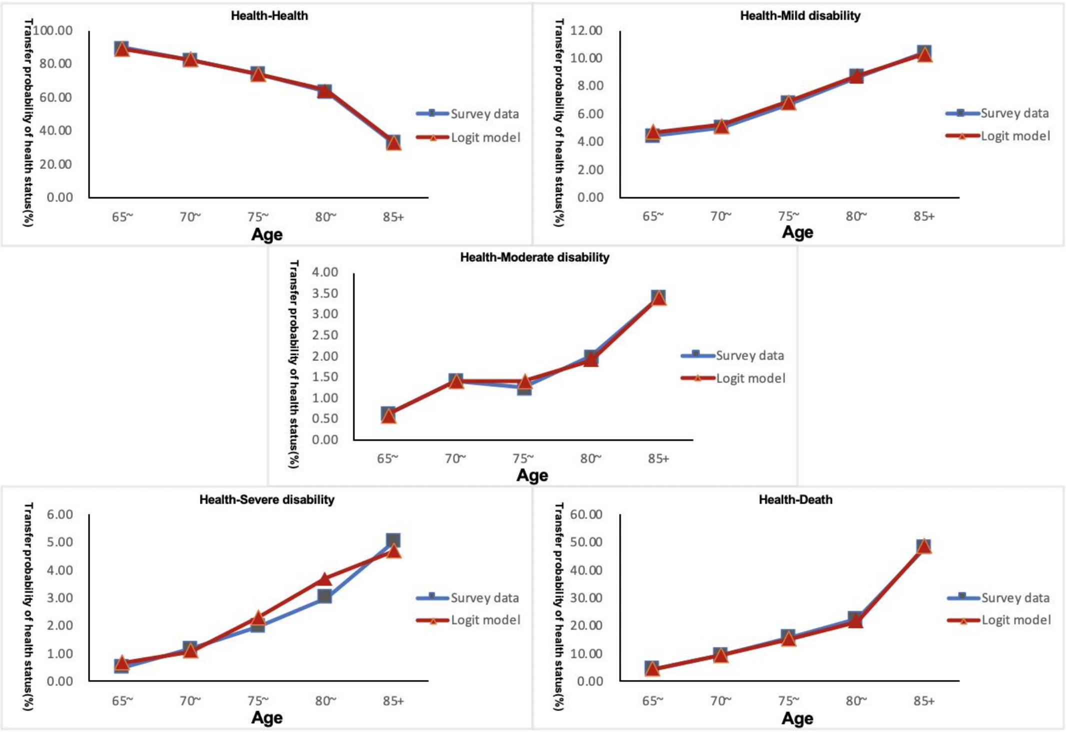

Figure S1. Distribution of health status from health to another health status

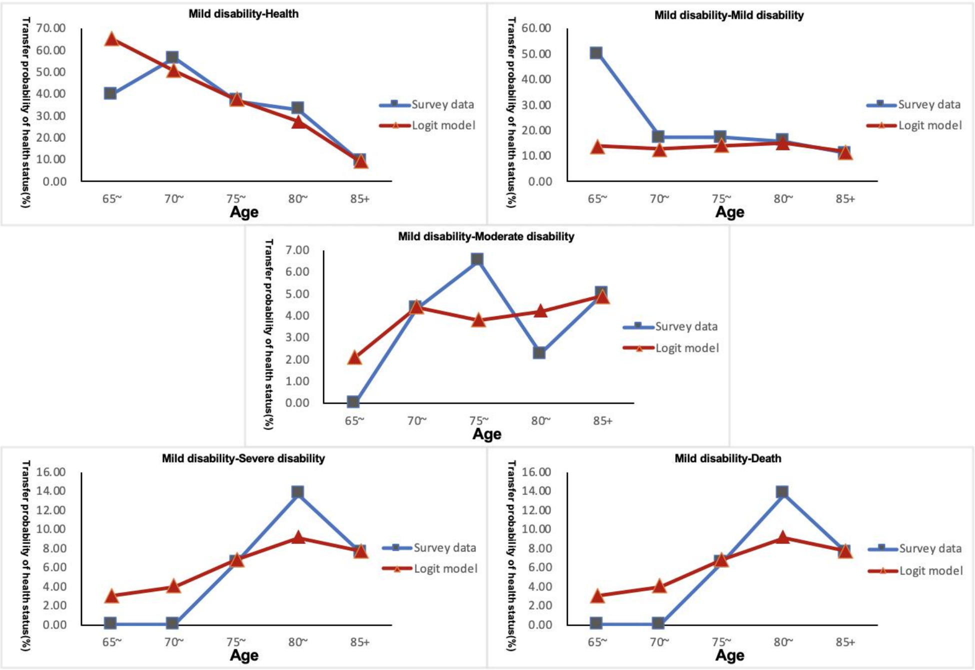

Figure S2. Distribution of health status from mild disability to another health status

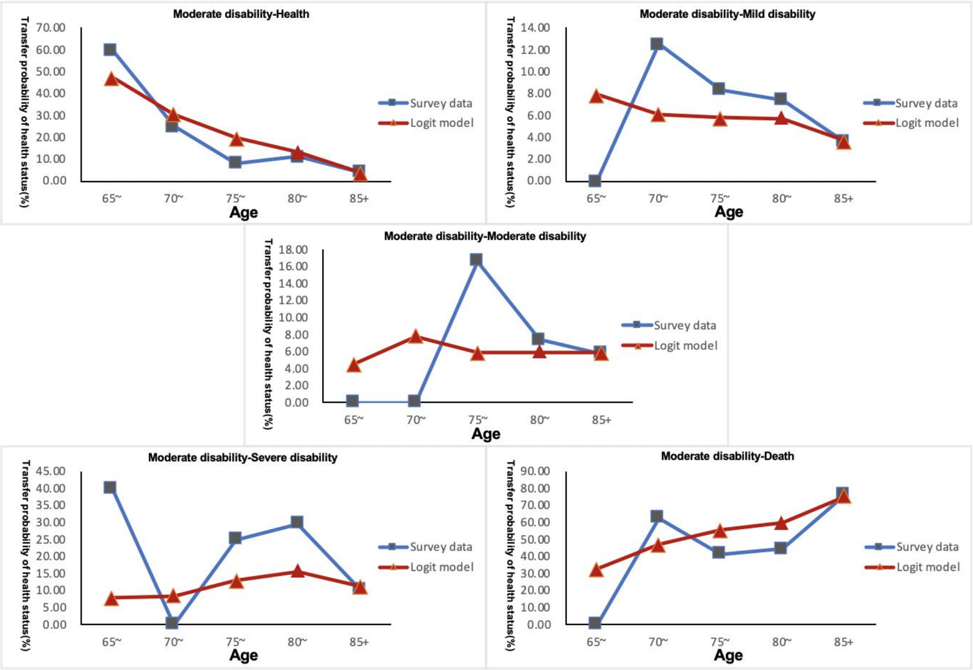

Figure S3. Distribution of health status from moderate disability to another health status

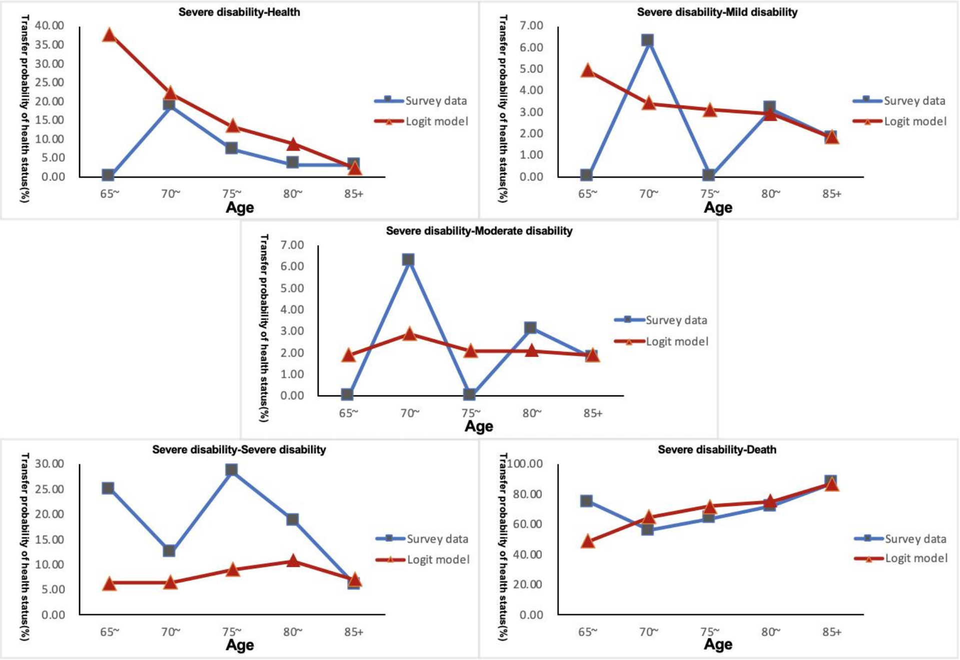

Figure S4. Distribution of health status from severe disability to another health status
